# Chlorine Dioxide-Iodide-Methyl Acetoacetate Oscillation Reaction Investigated by UV-Vis and Online FTIR Spectrophotometric Method

**DOI:** 10.1100/2012/918620

**Published:** 2012-02-01

**Authors:** Laishun Shi, Xiaomei Wang, Na Li, Jie Liu, Chunying Yan

**Affiliations:** School of Chemistry and Chemical Engineering, Shandong University, South Campus, Jinan 250061, China

## Abstract

In order to study the chemical oscillatory behavior and mechanism of a new chlorine dioxide-iodide ion-methyl acetoacetate reaction system, a series of experiments were done by using UV-Vis and online FTIR spectrophotometric method. The initial concentrations of methyl acetoacetate, chlorine dioxide, potassium iodide, and sulfuric acid and the pH value have great influence on the oscillation observed at wavelength of 289 nm. There is a preoscillatory or induction period, and the amplitude and the number of oscillations are associated with the initial concentration of reactants. The equations for the triiodide ion reaction rate changing with reaction time and the initial concentrations in the oscillation stage were obtained. Oscillation reaction can be accelerated by increasing temperature. The apparent activation energies in terms of the induction period and the oscillation period were 26.02 KJ/mol and 17.65 KJ/mol, respectively. The intermediates were detected by the online FTIR analysis. Based upon the experimental data in this work and in the literature, a plausible reaction mechanism was proposed for the oscillation reaction.

## 1. Introduction

Chemical oscillations are systems of chemicals that exhibit time-based fluctuations when they are far from equilibrium. The most studied chemical oscillation is the Belousov-Zhabotinsky (BZ) reaction, which is one of a class of reactions that serve as a classical example of nonequilibrium thermodynamics, resulting in the establishment of a nonlinear chemical oscillator. The detailed mechanism was first described by Field et al. (FKN) [1]. Field and Noyes constructed a simplified mathematical model, now known as the Oregonator [2]. Chlorite- and chlorine-dioxide-based chemical oscillations play an important role in “nonlinear chemistry.” Chlorite-iodide reaction has become, next to the Belousov-Zhabotinsky (BZ) reaction, perhaps the most widely studied reaction in nonlinear chemical dynamics. These studies have been concerned with the system nonlinear features, such as oscillations, bistability, stirring and premixing effects, spatial phenomena [3], and detailed mechanistic investigations.

Chlorine dioxide and sodium chlorite have close connection in the reaction. They can transfer each other in the whole pH ranges. The oxidation of iodide by chlorine dioxide was reported by Bray where it was used in the analytical determination of chlorine dioxide [4]. The kinetic study of the reaction between chlorine dioxide and potassium iodide in aqueous solution was investigated by Fukutomi and Gordon where two distinctly different rates were observed in the pH range 5.5–8.5 [5]. The first reaction corresponds to the rapid formation of the intermediate [ClO_2_I^−^]. The second corresponds to the decomposition of the intermediate, which results in the formation of ClO_2_^−^ and I_2_. The reaction between iodide ion and chlorite ion exhibits a remarkable variety of kinetic phenomena [6]. Responses to single and periodic pulse perturbations have been studied experimentally and numerically by Dolnik and Epstien for the reaction of chlorine dioxide and iodide ion in a stirred tank reactor [7]. Excitability with transient oscillations was obtained for perturbations by chlorine dioxide or chlorite, while stimulation by iodide produced no excitable response.

The dynamical behavior of the chlorine dioxide-iodide reaction has been studied in a system consisting of two continuous flow stirred tank reactors (CSTRs) [8]. By introducing an additional reactant to chlorite-iodide system that can react to regenerate the iodide consumed during each cycle of oscillation, De Kepper et al. constructed the chlorite-iodate-thiosulfate and the chlorite-iodide-malonic acid (MA) systems, which oscillate in a closed (batch) as well as in an open system [3, 9, 10]. The batch oscillation in the reaction of chlorine dioxide with iodine and malonic acid was studied by Lengyel et al. [11–13]. The modeling of the Turing structures in the chlorite-iodide-malonic acid-starch reaction system was also further investigated [14, 15].

Illumination of the chlorine dioxide-iodine-malonic acid reaction with visible light suppresses oscillations and shifts the steady state of the reaction to lower concentrations of iodide ions [16]. In the system with starch, illumination results in a strong decrease of the steady-state concentration of the triiodide-starch complex. They suggested a simple mechanism, in which iodine atoms produced by photodissociation of molecular iodine initiate reduction of chlorine dioxide to chlorite and oxidation of iodide ions to iodine. The oxidation of iodide ion by chlorine dioxide has been studied by stopped-flow techniques at *I* = 1.0 mol/L (NaClO_4_) [17]. A two-term rate law was confirmed for the reaction. Strier et al. and Szalai and De Kepper investigated the Turing patterns, spatial bistability, and front interactions in the [ClO_2_, I_2_, I^−^, CH_2_(COOH)_2_] reaction [18, 19]. The development of spiral pattern in a model representing chlorite-iodide-malonic acid reaction was investigated theoretically and numerically by Riaz and Ray [20]. A set of serially coupled flow reactors was modeled by Long et al. which contained chlorite-iodide oscillators [21]. By independently varying the reactor flow rates it is possible to produce oscillatory systems with differing periods where the ratio of the period of oscillation between reactors is always an integer value.

In previous papers we have studied the chlorine dioxide-iodine-malonic acid-sulfuric acid oscillation reaction [22] and sodium chlorite-iodine-ethyl acetoacetate oscillation reaction [23]. As is known, methyl acetoacetate (MAA) and malonic acid (MA) have similar properties in that both molecules contain an active CH_2_ group. By using methyl acetoacetate instead of malonic acid, oscillation should also occur for the system of ClO_2_-KI-MAA. In order to study the chlorine dioxide-iodide-methyl acetoacetate oscillator reaction better, it is necessary to investigate all of the factors that influence the reaction. Taking into account these factors, we found that oscillations can occur at 289 nm for the triiodide ion. Equations for the triiodide ion reaction rate changing with reaction time and the initial concentrations in the oscillation stage were obtained. The intermediates were detected by online FTIR analysis. A plausible reaction mechanism was proposed for the oscillation reaction.

## 2. Materials and Methods

### 2.1. Materials

Chlorine dioxide aqueous solutions were prepared from sodium chlorite and dilute sulfuric acid and were purified by bubbling of the resulting ClO_2_ through a 10% sodium chlorite aqueous solution to remove trace Cl_2_, then absorbing it in distilled water. Stock solutions of ClO_2_ were stored in darkness at 5°C. The ClO_2_ concentration was determined by the iodometric titration method. Potassium iodide solution was prepared by dissolving a certain amount of potassium iodide solid (AR) in a 500 mL measuring flask filled with distilled water. A methyl acetoacetate (MAA) aqueous solution was prepared by dissolving 3.00 mL methyl acetoacetate in a 500 mL measuring flask filled with distilled water. Citric acid-disodium hydrogen phosphate buffer solutions with different pHs were prepared from 0.2 mol/L Na_2_HPO_4_ solution and 0.1 mol/L citric acid solution. The sulfuric acid concentration was 0.04825 mol/L. All other chemicals were of the highest purity commercially available and were used as received.

### 2.2. Methods

The reaction was started by injecting a small volume of one of the reactants into a mixture containing the other components inside a spectrophotometric cell. The mixing time is about 2 or 3 s. Spectrophotometric measurements were performed in a TU-1800PC UV-Vis spectrophotometer (Beijing Puxi Tongyong Instrument Company, Beijing, China). A complete spectrum of the reaction mixture could be obtained at each second of reaction time. All measurements were performed at room temperature.

For the experiment of the temperature influence on the oscillation, a super electric-heated thermostatic water bath was used. The temperature of the water bath was set to 25°C, 30°C, 40°C, 45°C, and 50°C, respectively. The reactants were heated in the water bath. Following the above operation procedure, a group of oscillation curves about the absorbance of I_3_^−^ changing with reaction time at different temperatures could be obtained.

### 2.3. Online FTIR Analysis during the Oscillation Reaction

The online FTIR analysis was performed on a ReactIR 4000 spectrophotometer (Mettler-Toledo AutoChem, Inc., USA). FTIR spectra were recorded over the 648 to 4000 cm^−1^ wavenumber range at a resolution of 8 cm^−1^. The reaction conditions were fixed at [MAA]_0_ = 6.29 × 10^−3^ mol/L, [H_2_SO_4_]_0_ = 4.91 × 10^−3^ mol/L, [ClO_2_]_0_ = 7.60 × 10^−4^ mol/L, and [KI]_0_ = 3.62 × 10^−4^ mol/L.

## 3. Results and Discussion

### 3.1. Wavelength Selection

**Figure 1:**
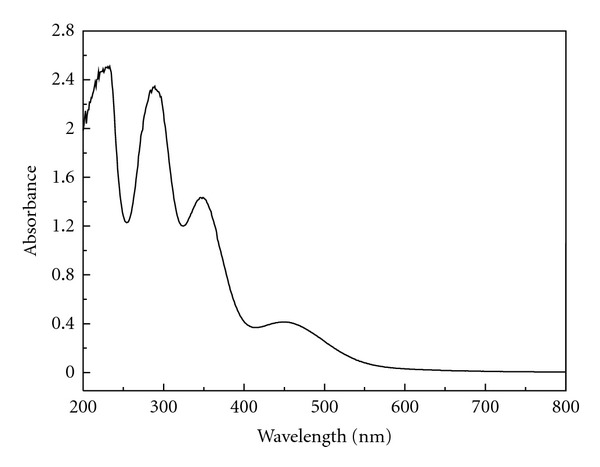
The UV-Vis spectrum of the reaction system.

Figure 1 gives the UV-Vis spectrum of ClO_2_-KI-MAA-H_2_SO_4_ reaction system at equilibrium. There are two absorption peaks at 289 nm and 350 nm, which should be the characteristic peaks of triiodide ion [24]. Therefore, the wavelength of 289 nm is selected in the following investigation.

### 3.2. Potassium Iodide Initial Concentration

**Figure 2:**
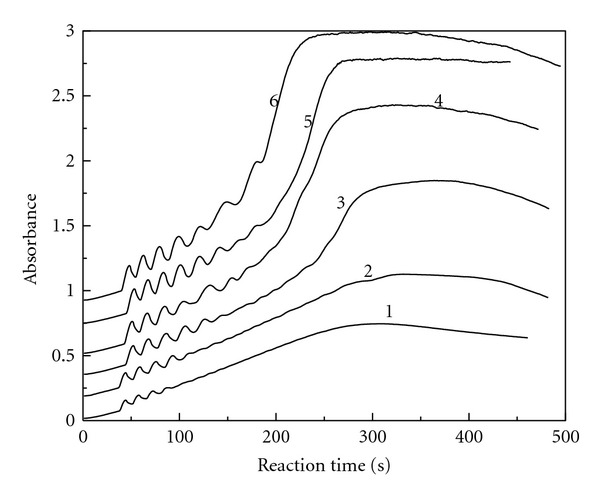
The absorbance versus reaction time at 289 nm for the triiodide ion. [ClO_2_]_0_ = 8.77 × 10^−4 ^ mol/L, [MAA]_0_ = 6.29 × 10^−3^ mol/L, [H_2_SO_4_]_0_ = 3.50 × 10^−3^ mol/L, [KI]_0_ = 2.72 × 10^−3^ mol/L (curve 1), 3.23 × 10^−3^ mol/L (curve 2), 3.40 × 10^−3^ mol/L (curve 3), 3.57 × 10^−3^ mol/L (curve 4), 3.74 × 10^−3^ mol/L (curve 5), 4.08 × 10^−3^ mol/L (curve 6).

**Figure 3:**
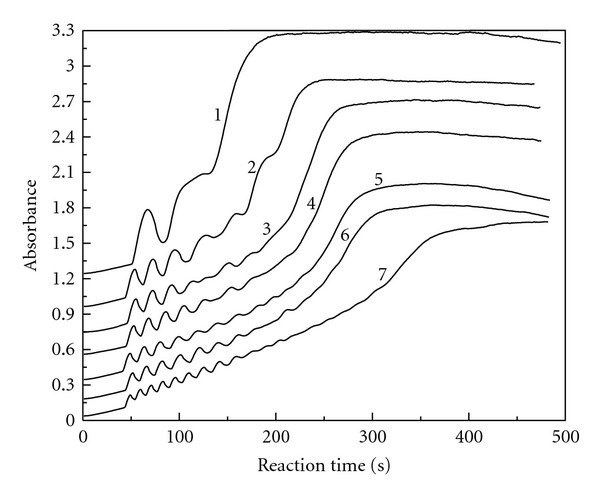
The absorbance versus reaction time at 289 nm for the triiodide ion: [MAA]_0_ = 6.29 × 10^−3^ mol/L, [H_2_SO_4_]_0_  = 3.50 × 10^−3^ mol/L, [KI]_0_ = 3.82 × 10^−3^ mol/L, [ClO_2_]_0_ = 8.43 × 10^−4^ mol/L (curve 1), 8.52 × 10^−4^ mol/L (curve 2), 8.76 × 10^−4^ mol/L (curve 3), 9.02 × 10^−4^ mol/L (curve 4), 9.11 × 10^−4^ mol/L (curve 5), 9.26 × 10^−4^ mol/L (curve 6), 9.51 × 10^−4^ mol/L (curve 7).

**Figure 4:**
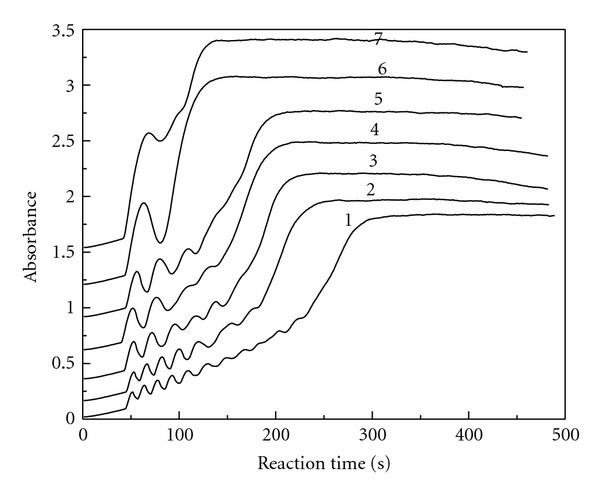
The absorbance versus reaction time at 289 nm for the triiodide ion: [ClO_2_]_0_ = 3.40 × 10^−4^ mol/L, [H_2_SO_4_]_0_ = 4.21 × 10^−3^ mol/L, [KI]_0_ = 3.82 × 10^−3^ mol/L, [MAA]_0_ = 5.66 × 10^−3^ mol/L (curve 1), 5.98 × 10^−3^ mol/L (curve 2), 6.29 × 10^−3^ mol/L (curve 3), 6.61 × 10^−3^ mol/L (curve 4), 6.92 × 10^−3^ mol/L (curve 5), 7.08 × 10^−3^ mol/L (curve 6), 7.24 × 10^−3^ mol/L (curve 7).

**Figure 5:**
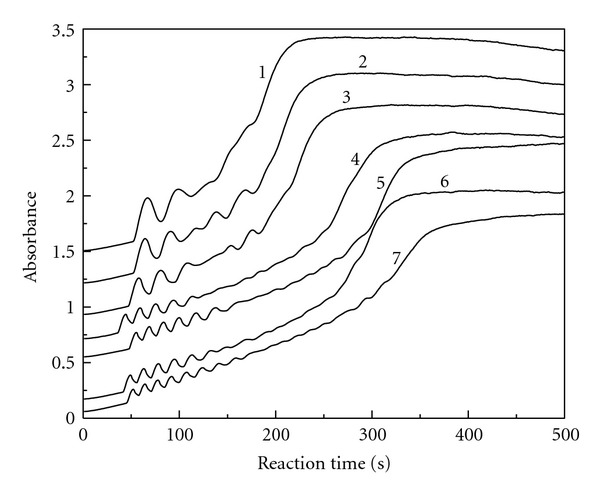
The absorbance versus reaction time at 289 nm for the triiodide ion: [MAA]_0_ = 6.29 × 10^−3^ mol/L, [KI]_0_ = 3.82 × 10^−3^ mol/L, [ClO_2_]_0_ = 3.86 × 10^−4^ mol/L, [H_2_SO_4_]_0 _= 3.50 × 10^−3^ mol/L (curve 1), 4.21 × 10^−3^ mol/L (curve 2), 4.91 × 10^-3 ^mol/L (curve 3), 5.61 × 10^−3^ mol/L (curve 4), 6.31 × 10^−3^ mol/L (curve 5), 7.01 × 10^−3^ mol/L (curve 6), 7.71 × 10^−3^ mol/L (curve 7).

**Figure 6:**
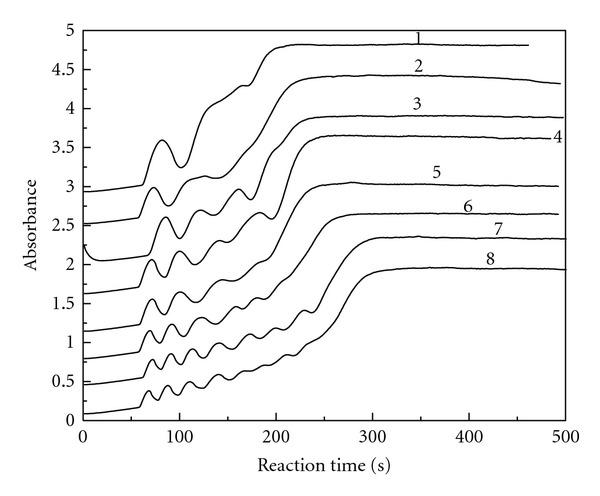
The absorbance versus reaction time at 289 nm for the triiodide ion: [ClO_2_]_0_/[KI]_0_ = 0.115, [MAA]_0_ = 6.29 × 10^−3 ^mol/L, [H_2_SO_4_]_0_ = 3.50 × 10^−3 ^mol/L, [ClO_2_]_0_ = 4.01 ×10^−4 ^mol/L, [KI]_0_ = 3.48 × 10^−3 ^mol/L (curve 1); [ClO_2_]_0_ = 4.21 × 10^−4 ^mol/L, [KI]_0_ = 3.65 × 10^−3 ^mol/L (curve2); [ClO_2_]_0_ = 4.40 × 10^−4 ^mol/L, [KI]_0_ = 3.82 × 10^−3 ^mol/L (curve 3); [ClO_2_]_0_ = 4.60 × 10^−4 ^mol/L, [KI]_0_ = 3.99 × 10^−3 ^mol/L (curve 4); [ClO_2_]_0_ = 4.70 × 10^−4 ^mol/L, [KI]_0_ = 4.08 × 10^−3 ^mol/L (curve 5); [ClO_2_]_0_ = 4.89 × 10^−4 ^mol/L, [KI]_0_ = 4.25 × 10^−3 ^mol/L (curve 6); [ClO_2_]_0_ = 5.09 × 10^−4 ^mol/L, [KI]_0_ = 4.42 × 10^−3 ^mol/L (curve 7); [ClO_2_]_0_ = 5.29 × 10^−4 ^mol/L, [KI]_0_ = 4.59 × 10^−3 ^mol/L (curve 8).

**Figure 7:**
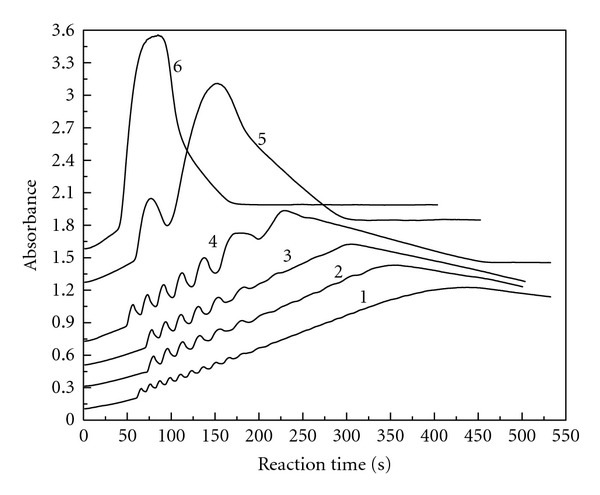
The absorbance versus reaction time at 289 nm for the triiodide ion: [MAA]_0_ = 6.29 × 10^−3 ^mol/L, [KI]_0_ = 3.82 × 10^−3 ^mol/L, [ClO_2_]_0_ = 5.96 × 10^−4 ^mol/L, pH = 2.2 (curve 1), 2.6 (curve 2), 3.0 (curve 3), 3.6 (curve 4), 4.0 (curve 5), 4.6 (curve 6).

**Figure 8:**
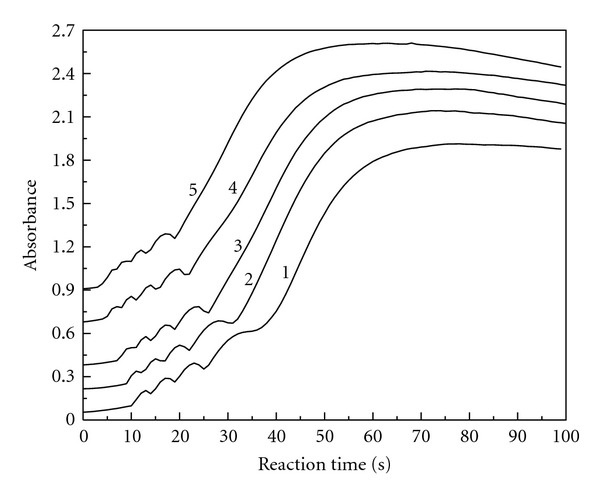
The absorbance versus reaction time at 289 nm for the triiodide ion: [ClO_2_]_0_ = 1.92 × 10^−3^ mol/L, [MAA]_0_ = 3.93 × 10^−3^ mol/L, [H_2_SO_4_]_0_ = 4.14 × 10^−3^ mol/L, [KI]_0_ = 3.22 × 10^−3^ mol/L, 25°C (curve 1), 30°C (curve 2), 35°C (curve 3), 45°C (curve 4), 55°C (curve 5).

We carried out the reaction of ClO_2_-KI-MAA in H_2_SO_4_ medium by measuring the absorbance change with reaction time at 289 nm. The reaction conditions were fixed at [MAA]_0_ = 6.29 × 10^−3^ mol/L, [ClO_2_]_0_ = 8.77 × 10^−4^ mol/L, and [H_2_SO_4_]_0_ = 3.50 × 10^−3^ mol/L by changing the initial concentration of potassium iodide.  Figure 2 gives the absorbance changes with reaction time at 289 nm for the triiodide ion. Successive curves are shifted up by an absorbance unit for better viewing, since without a shift the curves overlap. A similar treatment was also done in Figures 3 to 8. The oscillation phenomenon does not occur as long as the reactants are mixed. There is a preoscillatory or induction period. The oscillation phenomenon is obvious in the range of [KI]_0_ = 2.72 × 10^−4^ mol/L − 4.08 × 10^−4^ mol/L. The amplitude and the number of oscillations are associated with the initial concentration of potassium iodide, the amplitude increases with the initial concentration of potassium iodide, and also the number of oscillations increases.

We can differentiate the curves in Figure 2, and get the new curves of triiodide ion reaction rate (*ν*) change with reaction time. The new curves were processed by nonlinear curve fitting. We got the following equation (1) of triiodide ion reaction rate changing with reaction time and the initial concentration of potassium iodide in the oscillation stage:

(1) $$\begin{array}{cc} \nu & =\frac{\text{d}\left[{{\text{I}}_{3}}^{-}\right]}{\text{d}t}=P\;\mathrm{Sin}\left[\frac{2\pi \left(t-18\right)}{\text{T}}\right],\; \end{array}$$

where *P* represents the amplitude of the sine function. That is to say, it represents the amplitude of the oscillation reaction. The value of *P* is dependent on the initial concentration of potassium iodide. We observe that *P* follows the following equation:

(2) $$\begin{array}{cc} P & =\;-1.33\;\times \;10^{-2}\;+\;8.83\;{\left[\text{KI}\right]}_{0} \end{array}$$

with the linear correlation coefficient *r* = 0.969. The linear range of [KI]_0_  is 2.72 × 10^−3^ mol/L–4.08 × 10^−3^ mol/L. The range of amplitude *P* is 1.07 × 10^−2^–2.27 × 10^−2^, and it increases with the initial concentration of potassium iodide.

In (1), *T* represents the period of the sine function. That is to say, it represents the period of the oscillation reaction. The value of *T* is also dependent on the initial concentration of potassium iodide according to the following equation:

(3) $$\begin{array}{cc} T & =\;-10.4\;+\;8.02\;\times \;10^{3}{\left[\text{KI}\right]}_{0} \end{array}$$

with the linear correlation coefficient *r* = 0.982. The linear range of [KI]_0_ is 2.72 × 10^−3^ mol/L–4.08 × 10^−3^ mol/L. The range of period of oscillation *T* is 11.4*–*22.3 s, and it increases with the initial concentration of potassium iodide.

### 3.3. Chlorine Dioxide Initial Concentration

The reaction conditions were fixed at [KI]_0_ = 3.82 × 10^−3^ mol/L, [H_2_SO_4_]_0_ = 3.50 × 10^−3^ mol/L, and [MAA]_0_ = 6.29 × 10^−3^ mol/L with different [ClO_2_]_0_.  Figure 3 gives the absorbance changes with reaction time at 289 nm for the triiodide ion. As shown in Figure 3, the oscillation phenomenon does not occur as long as the reactants are mixed. There is a preoscillatory or induction period. The oscillation phenomenon is obvious in the range of 8.43 × 10^−4^ mol/L ⩽ [ClO_2_]_0_ ⩽ 9.51 × 10^−4^ mol/L. The amplitude and the number of oscillations are associated with the initial concentration of chlorine dioxide. As the initial concentration of chlorine dioxide increases, the amplitude decreases, while the number of oscillations increases.

We can also differentiate the curves in Figure 3, and get the new curves of triiodide ion reaction rate change with reaction time. Also, we got (1) of triiodide ion reaction rate change with reaction time and the initial concentration of chlorine dioxide in the oscillation stage.

In (1), the value of *P* is dependent on the initial concentration of chlorine dioxide according to the following equation:

(4) $$\begin{array}{cc} P & =0.103-96.9{\left[\text{Cl}{\text{O}}_{2}\right]}_{0} \end{array}$$

with the linear correlation coefficient *r* = 0.972. The linear range of [ClO_2_]_0_ is 8.43 × 10^−4^ mol/L–9.51 × 10^−4^ mol/L. The range of amplitude *P* is 2.13 × 10^−2^–1.08 × 10^−2^ and decreases with the initial concentration of chlorine dioxide.

In (1), the value of *T* is also dependent on the initial concentration of chlorine dioxide according to the following equation:

(5) $$\begin{array}{cc} T & =61.2-4.88\times 10^{4}{\left[\text{Cl}{\text{O}}_{2}\right]}_{0} \end{array}$$

with the linear correlation coefficient *r* = 0.980. The linear range of [ClO_2_]_0_  is 8.43 × 10^−4^ mol/L–9.51 × 10^−4^ mol/L. The range of period of oscillation *T* is 20.1–14.8 s and decreases with the initial concentration of chlorine dioxide.

### 3.4. MAA Initial Concentration

For the reaction of ClO_2_-KI-MAA in H_2_SO_4_ medium, the reaction condition was fixed at [KI]_0_ = 3.82 × 10^−3^ mol/L, [ClO_2_]_0_ = 3.40 × 10^−4^ mol/L, and [H_2_SO_4_]_0_ = 4.21 × 10^−3^ mol/L with different [MAA]_0_.  Figure 4 gives the absorbance changing with the reaction time at 289 nm for the triiodide ion. As shown in Figure 4, the oscillation phenomenon does not occur as long as the reactants are mixed. There is a preoscillatory or induction period. The oscillation phenomenon is obvious in the range of 5.66 × 10^−3^ mol/L ⩽ [MAA]_0_⩽ 7.24 × 10^−3^ mol/L. The amplitude and the number of oscillations are associated with the initial concentration of MAA. As the initial concentration of MAA increases, the amplitude also increases, but the number of oscillations decreases.

We can also differentiate the curves in Figure 4, and get the new curves of triiodide ion reaction rate change with reaction time. Also, we got (1) of triiodide ion reaction rate change with reaction time and the initial concentration of MAA in the oscillation stage.

In (1), the value of *P* is dependent on the initial concentration of MAA according to the following equation:

(6) $$\begin{array}{cc} P & =\;-7.42\;\times \;10^{-2}+15.6{\left[\text{MAA}\right]}_{0} \end{array}$$

with the linear correlation coefficient *r* = 0.975. The linear range of [MAA]_0_ is 5.66 × 10^−3^ mol/L–7.08 × 10^−3^ mol/L. The range of amplitude *P* is 1.41 × 10^−2^–3.62 × 10^−2^ and increases with the initial concentration of MAA.

In (1), the value of *T* is also dependent on the initial concentration of MAA according to the following equation:

(7) $$\begin{array}{cc} T & =32.6+8.41\times 10^{3}{\left[\text{MAA}\right]}_{0} \end{array}$$

with the linear correlation coefficient *r* = 0.998. The linear range of [MAA]_0_ is 5.66 × 10^−3^ mol/L–7.08 × 10^−3^ mol/L. The range of period of oscillation *T* is 15.0–26.9 s and increases with the initial concentration of MAA.

### 3.5. Sulfuric Acid Initial Concentration

The reaction conditions were fixed at [MAA]_0_ = 6.29 × 10^−3^ mol/L, [ClO_2_]_0_ = 3.86 × 10^-4 ^mol/L, and [KI]_0_ = 3.82 × 10^−3^ mol/L while the initial sulfuric acid concentration was varied.  Figure 5 gives the absorbance change with reaction time at 289 nm for the triiodide ion. As shown in Figure 5, the oscillation phenomenon does not occur as long as the reactants are mixed. There is a preoscillatory or induction period. The oscillation phenomenon is obvious in the whole range of [H_2_SO_4_]_0_ = 3.50 × 10^−3^ mol/L–7.71 × 10^−3^ mol/L. The amplitude is large at the beginning stage then decreases with reaction time. Finally, the oscillation ceases suddenly. The number of oscillations increases with the increase of the initial concentration of sulfuric acid.

Differentiation of the curve in Figure 5 yields new curves and we can get (1) for the reaction rate changes with reaction time and the initial concentration of H_2_SO_4_ in the oscillation stage.

In (1), the value of *P* is dependent on the initial concentration of H_2_SO_4_ according to the following equation:

(8) $$\begin{array}{cc} P & =\;3.16\;\times \;10^{-2}\;-\;2.76{\left[{\text{H}}_{2}{\text{SO}}_{4}\right]}_{0} \end{array}$$

with the linear correlation coefficient *r* = 0.975. The linear range of [H_2_SO_4_]_0_ is 3.50 × 10^−3^ mol/L–7.71 × 10^−3^ mol/L. The range of amplitude *P* is 2.19 × 10^−2^–1.03 × 10^−2^, and it decreases with the initial concentration of H_2_SO_4_.

In (1), the value of *T* is also dependent on the initial concentration of H_2_SO_4_ according to the following equation:

(9) $$\begin{array}{cc} T\; & =39.0-3.40\times 10^{3}{\left[{\text{H}}_{2}{\text{SO}}_{4}\right]}_{0} \end{array}$$

with the linear correlation coefficient *r* = 0.963. The linear range of [H_2_SO_4_]_0_ is 3.50 × 10^−3^ mol/L–7.71 × 10^−3^ mol/L. The range of period of oscillation *T* is 27.1–12.8 s, and it decreases with the initial concentration of H_2_SO_4_.

### 3.6. The Influence of Dilution

In order to investigate the influence of the reactant concentrations on the oscillation, experiments were carried out by fixing [H_2_SO_4_]_0_ = 3.50 × 10^−3 ^mol/L and [MAA]_0_ = 6.29 × 10^−3 ^mol/L keeping the molar ratio of [ClO_2_]_0_/[KI]_0_ = 0.115, and changing the initial concentrations of chlorine dioxide and potassium iodide at the same time.  Figure 6 gives the absorbance change with reaction time at 289 nm for the triiodide ion at different initial concentrations of chlorine dioxide and potassium iodide. As shown in Figure 6, the oscillation phenomenon occurs in the whole range of [ClO_2_]_0_ = 4.01 × 10^−4 ^mol/L–5.29 × 10^−4 ^mol/L and [KI]_0_ = 3.48 × 10^−3 ^mol/L–4.59 × 10^−3 ^mol/L. There is a preoscillatory or induction period; when the initial concentrations of chlorine dioxide and potassium iodide are higher, the oscillation phenomenon is more obvious and regular. The amplitude decreases and the number of oscillations increases along with the increase of the initial concentration of chlorine dioxide and potassium iodide.

### 3.7. pH Value

The reaction conditions were fixed at [MAA]_0_ = 6.29 × 10^−3 ^mol/L, [ClO_2_]_0_ = 5.96 × 10^−4 ^mol/L, and [KI]_0_ = 3.82 × 10^−3 ^mol/L while the pH value was varied.  Figure 7 gives the absorbance change with reaction time at 289 nm for the triiodide ion. As shown in Figure 7, the oscillation phenomenon does not occur as long as the reactants are mixed. There is a preoscillatory or induction period. The oscillation phenomenon is obvious in the whole range of pH = 2.2–4.0. At pH values above 4.0, it is difficult to observe the oscillation (see curve 6). The amplitude is large at the beginning stage then decreases with reaction time. Finally, the oscillation ceases suddenly. The amplitude and the number of oscillations are associated with the pH value. As the pH value increases, the amplitude increases, but the number of oscillations decreases.

### 3.8. The Influence of Temperature on the Oscillation

Figure 8 gives the absorbance changes with reaction time at 289 nm for the triiodide ion. The reaction conditions were fixed at [ClO_2_]_0_ = 1.92 × 10^−3^ mol/L, [MAA]_0_ = 3.93 × 10^−3^ mol/L, [H_2_SO_4_]_0_ = 4.14 × 10^−3^ mol/L, and [KI]_0_ = 3.22 × 10^−3^ mol/L. Five oscillation curves were obtained in the temperature range of 25°C to 55°C. As shown in Figure 8, both the induction period and the oscillation period decrease with the increasing of reaction temperature. The experiments show that oscillation reaction can be accelerated by increasing reaction temperature, but oscillation life is shortened.

According to the Arrhenius equation

(10) $$\begin{array}{cc} k & =A\cdot e^{-Ea/RT}, \end{array}$$

taking the logarithm of (10)

(11) $$\begin{array}{cc} \ln k & =\;\ln A-\left(\frac{Ea}{RT}\right), \end{array}$$

ln*k* ~ (1/*T*) should be linear relationship. The slope is −*Ea*/*R*. Thus, the activation energy *Ea* can be calculated.

The aim of the experiment is to study the apparent activation energy (*Eu*) in terms of the induction period and the apparent activation energy (*Ez*) in terms of the oscillation period in the oscillation reaction. The induction time (*tu*) and the oscillation period (*tz*) can be obtained from Figure 8. Therefore, (11) can be written as

(12) $$\begin{array}{cc} \ln\left(\frac{1}{tu}\right) & =\;\ln A-\left(\frac{Eu}{RT}\right),\\ \ln\left(\frac{1}{tz}\right) & =\;\ln A-\left(\frac{Ez}{RT}\right). \end{array}$$

ln (1/*tu*) ~1/*T* and ln (1/*tz*) ~1/*T* should be linear relationships, respectively. Thus, the apparent activation energies *Eu* and *Ez* can be calculated, respectively.

**Figure 9:**
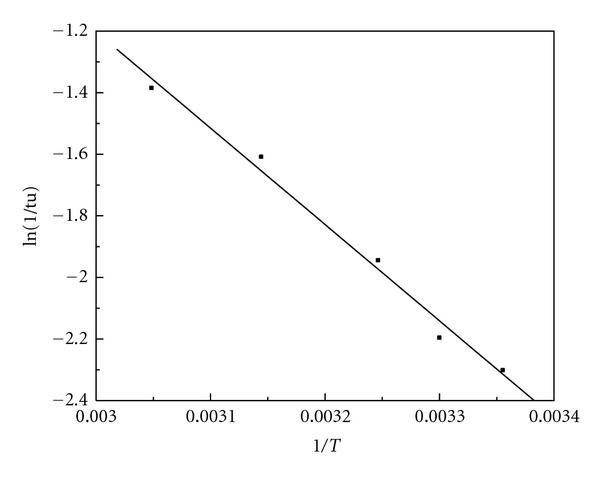
The curve of ln (1/*tu*) versus 1/*T. *

**Figure 10:**
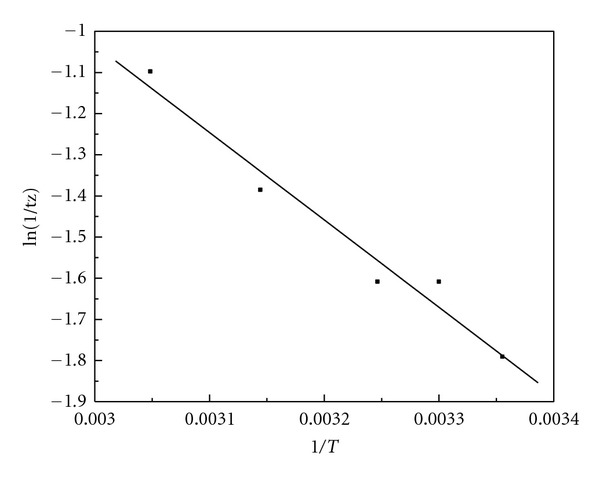
The curve of ln (1/*tz*) versus 1/*T. *

Table 1 gives the induction time (*tu*) and the oscillation period (*tz*) at various reaction temperatures.  Figure 9 shows the linear fitting of ln (1/*tu*) versus 1/*T*.  Figure 10 shows the linear fitting of ln (1/*tz*) versus 1/*T*. Therefore, (12) can be written as

(13) $$\begin{array}{cc} \ln\left(\frac{1}{tu}\right) & =\;8.185-3129\left(\frac{1}{T}\right), \end{array}$$

(14) $$\begin{array}{cc} \ln\left(\frac{1}{tz}\right) & =\;5.335-2123\left(\frac{1}{T}\right). \end{array}$$

The linear correlation coefficient of (13) is 0.994. By using the slope of (13), we can obtain the apparent activation energy (*Eu*) in terms of the induction period is 26.02 KJ/mol. The linear correlation coefficient of (14) is 0.982. By using the slope of (14), we can get that the apparent activation energy (*Ez*) in terms of the oscillation period is 17.65 KJ/mol. The apparent activation energy of the oscillation reaction is relatively small.

### 3.9. Online FTIR Analysis during the Oscillation Reaction

**Figure 11:**
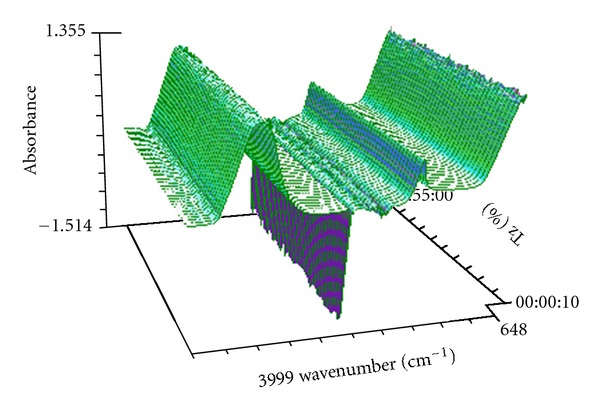
The 3D online infrared spectrum during the oscillation reaction.

Figure 11 gives the 3D online infrared spectrum during the oscillation reaction. In Figure 11, the strong absorption near 3307 cm^−1^ is assigned to the O–H stretching of water. The strong absorption near 1652 cm^−1^ is assigned to the H–O–H bending of water.

**Figure 12:**
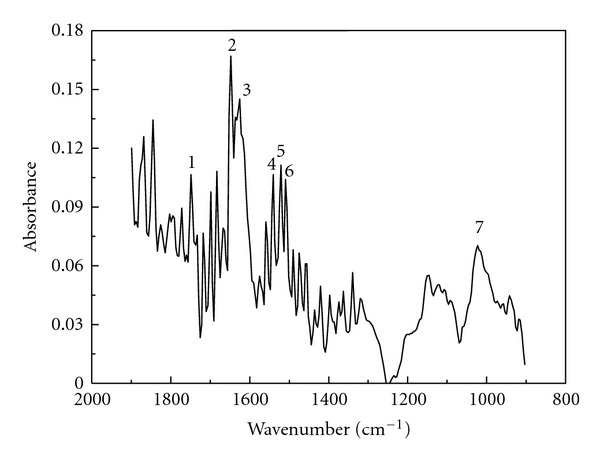
FTIR spectrum of intermediate 1.

Two intermediates were detected automatically during the online FTIR analysis, and their FTIR spectra were obtained. In the FTIR spectrum of intermediate 1 (Figure 12), the absorption near 1747 cm^−1^ (peak 1) is assigned to the C=O stretching. The absorption near 1652 cm^−1^ (peak 2) and 1629 cm^−1^ (peak 3) is assigned to the C=O stretching of an enol isomer of keto-enol tautomerism. The absorptions near 1544, 1525, and 1513 cm^−1^ (peak 4–6) are assigned to the C=C stretching of the enol isomer of keto-enol tautomerism. The absorption near 1031 cm^−1^ (peak 7) is assigned to the O–H in-plane deformation in the enol isomer of keto-enol tautomerism. In the FTIR spectrum of intermediate 2 (the figure is omitted), the absorption near 1652 cm^−1^ is assigned to the C=O stretching of the iodine-substituted keto isomer of keto-enol tautomerism.

**Figure 13:**
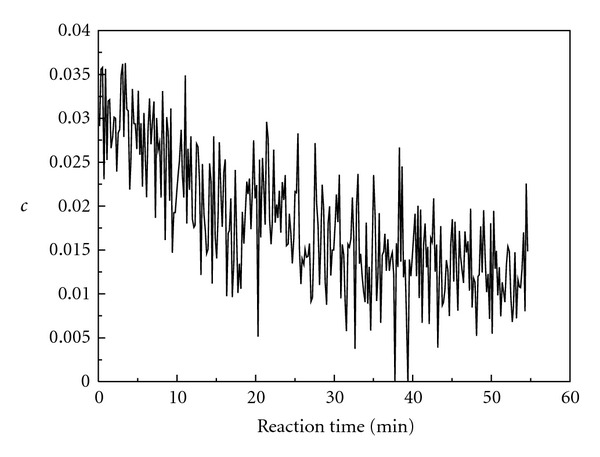
The curve of intermediate 1 relative concentration versus reaction time.

**Figure 14:**
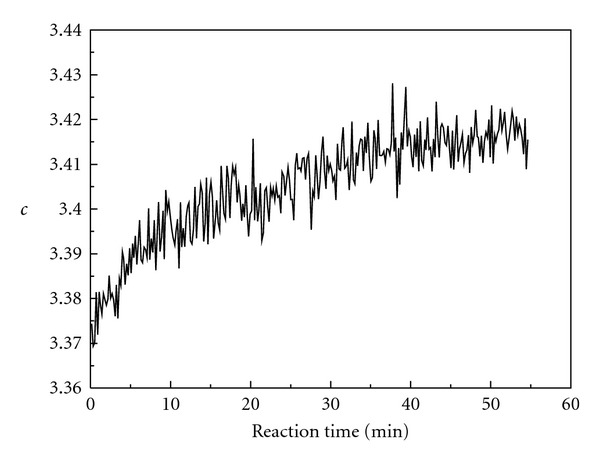
The curve of intermediate 2 relative concentration versus reaction time.

 Figures 13 and 14 give the relative concentrations (*c*) of intermediates 1 and 2 versus reaction time. As shown in Figure 13, the relative concentration of intermediate 1 decreases with reaction time. That is to say, the concentration of enol isomer decreases during the oscillation reaction. As shown in Figure 14, the relative concentration of intermediate 2 increases with reaction time. That is to say, the concentration of iodine-substituted keto isomer increases during the oscillation reaction.

According to the literature [12] the behavior of ClO_2_-I^−^-MAA chemical oscillatory reaction system can be modeled by a simple scheme consisting of three component reactions. The reactions should be as follows

(15) $$\begin{array}{cc} & {\text{ClO}}_{2}+{\text{I}}^{-}\to {{\text{ClO}}_{2}}^{-}+\;1/2{\text{I}}_{2} \end{array}$$

(16) $$\begin{array}{cc} & {{\text{ClO}}_{2}}^{-}+4{\text{I}}^{-}+4{\text{H}}^{+}\to \;{\text{Cl}}^{-}+2{\text{I}}_{2}+2{\text{H}}_{2}\text{O} \end{array}$$

(17) $$\begin{array}{cc} & {\text{CH}}_{3}{\text{COCH}}_{2}{\text{COOCH}}_{3}+{\text{I}}_{2}\\ & \;\to {\text{CH}}_{3}{\text{COCHICOOCH}}_{3}+{\text{I}}^{-}+{\text{H}}^{+} \end{array}$$

For reaction (17), we proposed a scheme as shown in reactions (R4)–(R6) based upon the concentration changing trends of the two intermediates in Figures 13 and 14 and in consideration literature [25]. In aqueous solutions putting into the base shown in reaction (R5) may be water or halide ions which are sufficiently basic to act as shown in [26]. One possibility is that either reaction (R5) or (R6) can be a rate-determining step: 


(R4)
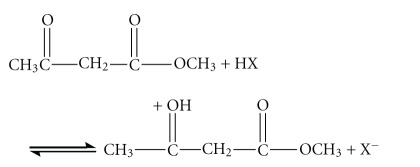

(R5)
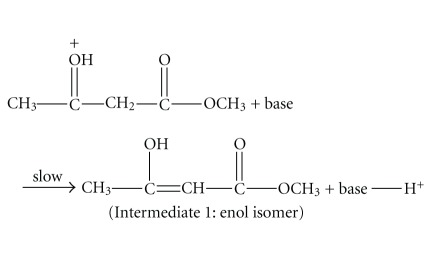

(R6)
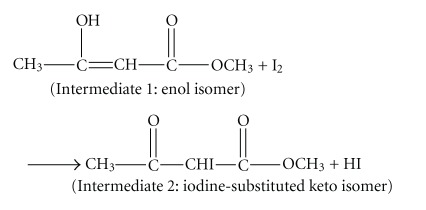



## 4. Conclusion

The initial concentrations of methyl acetoacetate, chlorine dioxide, potassium iodide, and sulfuric acid have great influence on the oscillation at 289 nm. There is a preoscillatory or induction period. Equations for the triiodide ion reaction rate changes with reaction time and the initial concentrations in the oscillation stage were obtained. Oscillation reaction can be accelerated by increasing temperature. The apparent activation energy in terms of the induction period or the oscillation period was calculated. The intermediates were detected by the online FTIR analysis. Based upon the experimental data in this work and in the literature, a plausible reaction mechanism was proposed for the oscillation reaction.

## Figures and Tables

**Table 1:** The induction time (*tu*) and the oscillation period (*tz*) at various reaction temperatures.

Reaction temperature (K)	298.0	303.0	308.0	318.0	328.0
*tu* (s)	10.0	9.0	7.0	5.0	4.0
*tz* (s)	6.0	5.0	5.0	4.0	3.0
